# Chromatin architecture may dictate the target site for DMC1, but not for RAD51, during homologous pairing

**DOI:** 10.1038/srep24228

**Published:** 2016-04-07

**Authors:** Wataru Kobayashi, Motoki Takaku, Shinichi Machida, Hiroaki Tachiwana, Kazumitsu Maehara, Yasuyuki Ohkawa, Hitoshi Kurumizaka

**Affiliations:** 1 Laboratory of Structural Biology, Graduate School of Advanced Science & Engineering, Waseda University, 2-2 Wakamatsu-cho, Shinjuku-ku, Tokyo 162-8480, Japan; 2 Division of Transcriptomics, Medical Institute of Bioregulation, Kyushu University, Fukuoka, 812-8582, Japan; 3 Institute for Medical-oriented Structural Biology, Waseda University, 2-2 Wakamatsu-cho, Shinjuku-ku, Tokyo 162-8480, Japan

## Abstract

In eukaryotes, genomic DNA is compacted as chromatin, in which histones and DNA form the nucleosome as the basic unit. DMC1 and RAD51 are essential eukaryotic recombinases that mediate homologous chromosome pairing during homologous recombination. However, the means by which these two recombinases distinctly function in chromatin have remained elusive. Here we found that, in chromatin, the human DMC1-single-stranded DNA complex bypasses binding to the nucleosome, and preferentially promotes homologous pairing at the nucleosome-depleted regions. Consistently, DMC1 forms ternary complex recombination intermediates with the nucleosome-free DNA or the nucleosome-depleted DNA region. Surprisingly, removal of the histone tails improperly enhances the nucleosome binding by DMC1. In contrast, RAD51 does not specifically target the nucleosome-depleted region in chromatin. These are the first demonstrations that the chromatin architecture specifies the sites to promote the homologous recombination reaction by DMC1, but not by RAD51.

In meiosis, two rounds of cell division, meiotic cell divisions I and II, occur with a single round of DNA replication, thus producing gametes containing a haploid genome^1^. During meiotic cell division I, the crossover type of homologous recombination (meiotic homologous recombination) occurs between homologous chromosomes. Consequently, a physical connection between homologous chromosomes is formed, as a chiasma^1^. The failure of chiasma formation causes chromosome non-disjunction, which is a major source of fetal aneuploidy, indicating that the crossover recombination producing the chiasma plays a crucial role to ensure accurate chromosome segregation during meiotic cell division I. Meiotic homologous recombination occurs in the chromatin loops of the synaptonemal complex, a macro protein-DNA complex, which is formed between homologous chromosomes containing two pairs of sister chromatids^1^.

Homologous recombination is usually initiated by the induction of DNA double strand breaks (DSBs)^2^. In mitotic cells, DSBs are randomly induced in non-defined chromosome regions by ionizing radiation, replication failure, and DNA damaging chemicals. In contrast, in meiosis, homologous recombination is initiated by programmed DSBs, which are actively induced by the topoisomerase-family enzyme, SPO11, at defined chromosome loci^3^,^4^,^5^. In eukaryotic chromosomes, genomic DNA is wrapped around histone octamers composed of the four core histones, H2A, H2B, H3, and H4, and forms nucleosomes accommodating about 150 base-pairs of DNA, as the basic unit of chromatin^6^. Nucleosome formation generally restricts the accessibility of proteins, including SPO11, to DNA. Interestingly, meiotic DSBs are reportedly introduced on the chromatin loop regions that transiently interact with the lateral elements of the synaptonemal complex^7^,^8^,^9^,^10^, suggesting that these chromatin regions may contain nucleosome-depleted regions.

Some of these DSB sites are hot spots for meiotic homologous recombination between homologous chromosomes. In yeasts, these meiotic recombination hot spots are generally open chromatin structures containing nucleosome-depleted regions, where the SPO11 that is transiently associated with the lateral elements can access the DNA^10^,^11^,^12^,^13^,^14^. In mouse chromosomes, the recombination hot spots primarily possess open chromatin configurations^15^,^16^,^17^,^18^. However, in mice, the presence of a well-positioned nucleosome at the meiotic DSB site before the DSB induction was also reported^19^. This contradiction may be reconciled, if the nucleosome-depleted regions are actively produced at the DSB sites. In this context, Baker *et al*.^20^ studied two inbred mouse strains, and reported that nucleosome-depletion is actually established at recombination hot spots by the function of PRDM9, which tri-methylates the Lys4 residue of histone H3 (H3K4me3). Therefore, the open chromatin structure containing nucleosome-depleted regions may be a common architecture of meiotic recombination hot spots.

After DSB formation, single-stranded DNA (ssDNA) regions are produced at the DSB sites. The ssDNA region then invades the homologous double-stranded DNA (dsDNA), and new Watson-Crick base pairs are formed between the invading strand and the complementary strand of the parental dsDNA. This homology-directed strand invasion, called “homologous pairing”, is the central step for homologous recombination, and the eukaryotic recombinases, DMC1 and RAD51, catalyze homologous pairing between the two DNA molecules^21^,^22^,^23^,^24^. To promote homologous pairing, DMC1 and RAD51 first bind to the ssDNA region, and form filamentous nucleoprotein complexes. The DMC1-ssDNA or RAD51-ssDNA complex is then bound to the dsDNA target, and the homologous DNA sequences are aligned within the ternary complex containing ssDNA, dsDNA, and DMC1 or RAD51.

In mammals, DMC1 and RAD51 share high amino acid sequence similarity (more than 50%), and biochemical studies revealed that both DMC1 and RAD51 efficiently promote homologous pairing between ssDNA and dsDNA^21^,^22^,^23^,^24^. However, there are obvious differences in their expression profiles. RAD51 is expressed in both mitotic and meiotic cells^25^,^26^, but DMC1 is only produced in meiotic cells^27^,^28^. Genetic studies indicated that *RAD51−/−* mice exhibited embryonic lethality^29^, but *DMC1−/−* mice grew normally^30^,^31^. However, *DMC1−/−* mice are completely sterile, because of severe defects in meiotic chromosome segregation due to the failure of homologous chromosome pairing during meiotic cell division I^30^,^31^. Interestingly, previous studies with the *Arabidopsis thaliana* RAD51-GFP fusion protein and the *Saccharomyces cerevisiae* Rad51 II3A mutant (in which the Arg188, Lys361, and Lys371 residues were replaced by Ala) revealed that these engineered RAD51 proteins, which are defective in mitotic DSB repair, are proficient in meiotic homologous recombination in the presence of DMC1^32^,^33^. Consistently, these engineered RAD51 proteins were found to be defective in their homologous-pairing activity, but they retained the activity to stimulate the DMC1-mediated homologous pairing *in vitro*^32^,^34^. These findings indicated that DMC1 plays a central role as a catalyst in meiotic homologous pairing, with RAD51 as an activator. However, the mechanism by which DMC1 functions in the meiotic homologous pairing between the chromatin loops remains elusive.

In the present study, we found that the DMC1-ssDNA complex promoted homologous pairing in the chromatin context by targeting the nucleosome-depleted homologous spots, but the RAD51-ssDNA complex did not. Consistently, the DMC1-ssDNA complex barely bound to nucleosomes, and specifically targeted the nucleosome-depleted dsDNA region. In contrast, the RAD51-ssDNA complex was strongly trapped by nucleosomes, and therefore did not target the nucleosome-depleted dsDNA. Surprisingly, the removal of the histone tails enhanced the nucleosome binding by DMC1, suggesting that the histone tails may restrict the DMC1-nucleosome interaction. These results are the first demonstration that the nucleosome specifically attenuates RAD51, while the DMC1-mediated homologous pairing occurs in the nucleosome-depleted DNA regions of chromatin.

## Results

### Homologous pairing by DMC1 or RAD51 in chromatin containing nucleosome-depleted dsDNA regions

**Figure 1:**
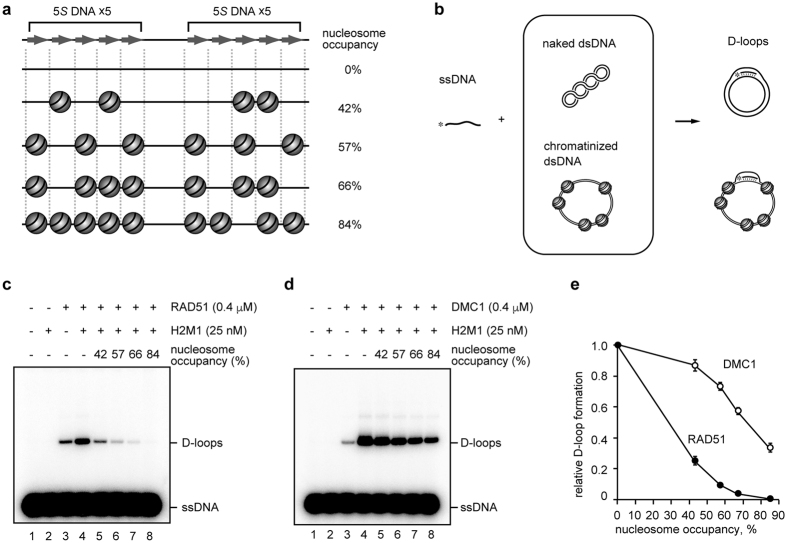
Homologous pairing in chromatin. (**a**) Schematic representation of the nucleosomal dsDNA. The light gray arrows indicate the homologous regions of the 5*S* ssDNA 70-mer (top). (**b**) Scheme of the D-loop formation assay. Asterisks indicate the ^32^P-labeled 5′-end of the ssDNA. (**c,d**) D-loop formation on the 5*S* DNA sequences with RAD51 or DMC1. RAD51 (0.4 μM) or DMC1 (0.4 μM) was incubated with the 5*S* ssDNA 70-mer (final 1 μM in nucleotides). The reactions were conducted in the presence of HOP2-MND1 (denoted as H2M1). The nucleosome occupancies on the 5*S* DNA repeats were 0% (lanes 1–4), 42% (lane 5), 57% (lane 6), 66% (lane 7), and 84% (lane 8). Panels (**c**,**d**) represent experiments with RAD51 and DMC1, respectively. (**e**) Graphic representation of the experiments shown in panels (**c**,**d**). The amounts of D-loop formation relative to RAD51 or DMC1 with HOP2-MND1 (lane 4 of panels (**c**,**d**)) are plotted against the nucleosome occupancies. The average values of three independent experiments are shown with the SD values.

To compare the DMC1 and RAD51 homologous pairing activities in chromatinized dsDNA templates, we reconstituted nucleosome arrays with different nucleosome occupancies, and performed the D-loop formation assay, which is the standard assay for homologous pairing (Fig. 1a,b). Human DMC1, RAD51, and core histones were purified as recombinant proteins (Supplementary Fig. 1). We used plasmid DNA containing ten tandem repeats of 5*S* DNA, which is known to form positioned nucleosomes (Fig. 1a). Therefore, ten positioned nucleosomes will be formed in the region containing the ten 5*S* DNAs, if the nucleosome occupancy of the reconstituted chromatin is 100%. In this study, we prepared four nucleosome arrays, in which the nucleosome occupancies on the 5*S* DNA sequences were 42%, 57%, 66%, and 84%, respectively (Fig. 1a and Supplementary Fig. 2).

We performed the D-loop formation assay with the ssDNA 70-mer containing the 5*S* DNA sequence and each reconstituted nucleosome array (Fig. 1b), in the presence of the HOP2-MND1 complex, which is a prominent activator for both RAD51- and DMC1-mediated homologous pairing^35^,^36^,^37^,^38^,^39^. The RAD51-ssDNA complex requires RAD54, a nucleosome remodeler, to promote homologous pairing with the nucleosomal dsDNA^40^,^41^,^42^. However, for the direct comparison of the homologous-pairing activities between DMC1 and RAD51 in chromatin, we performed the D-loop formation assay in the absence of RAD54.

As compared to the experiments with the naked dsDNA, the RAD51-mediated homologous pairing was markedly inhibited in the presence of nucleosomes (Fig. 1c,e). Interestingly, RAD51 exhibited very low homologous pairing activity with the 42% nucleosome array, although about six homologous 5*S* DNA sequences were present as the nucleosome-depleted dsDNA regions (Fig. 1c, lane 5, and 1e). This suggested that the RAD51-ssDNA complex was trapped by the nucleosome, and could not overcome the nucleosome barrier without the nucleosome remodeler, RAD54. In contrast, we found that DMC1 efficiently and equivalently promoted homologous pairing with the 42% nucleosome array and the naked dsDNA (Fig. 1d, lane 5, and 1e). The DMC1-mediated homologous pairing was somewhat inhibited in the 57%, 66%, and 84% nucleosome arrays, but was still more efficient than the RAD51-mediated homologous pairing (Fig. 1c–e). These results suggested that the nucleosome formation directly inhibits the RAD51 activity, but not the DMC1 activity, in homologous pairing.

### The presence of nucleosomes minimally affects the DMC1 activity, but substantially inhibits the RAD51-mediated homologous pairing

**Figure 2:**
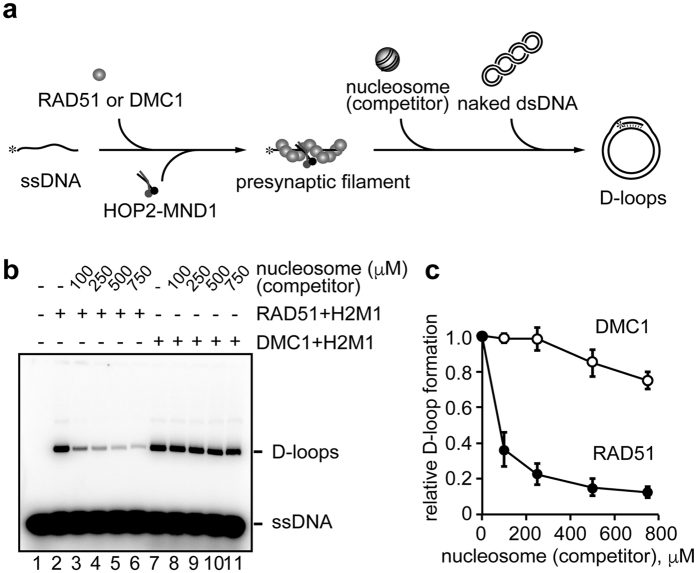
Competitive homologous-pairing assay. (**a**) Scheme of the competitive homologous-pairing assay. Asterisks indicate the ^32^P-labeled 5′-end of the 5*S* ssDNA 70-mer. (**b**) The D-loop formation assay on the 5*S* DNA sequences. RAD51 (0.4 μM) or DMC1 (0.4 μM) was incubated with the 5*S* ssDNA 70-mer (final 1 μM in nucleotides). The reactions were conducted in the presence of HOP2-MND1 (denoted as H2M1). The reaction was initiated by adding the naked dsDNA (final 30 μM in nucleotides) in the presence of the competitor nucleosome. The nucleosome concentrations are indicated at the top of the panel. After a 10 min incubation, the reaction was stopped by SDS and proteinase K, and the reaction products were separated by agarose gel electrophoresis. (**c**) Graphic representation of the experiments shown in panel (**b**). The amounts of D-loop formation relative to RAD51 or DMC1 with HOP2-MND1 (lanes 2 and 7 of panel (**b**)) are plotted against the nucleosome concentrations. The average values of four independent experiments are shown with the SD values.

To study the effect of the presence of nucleosomes on homologous pairing, we next tested whether the reconstituted nucleosomes added to the reaction mixture affect the D-loop formation by RAD51 and DMC1 in *trans* (Fig. 2a). In this assay, naked supercoiled DNA was used as the target dsDNA substrate (Fig. 2a). The nucleosome titration experiments revealed that the RAD51-mediated homologous pairing was markedly inhibited by nucleosomes (Fig. 2b, lanes 3–6, and 2c). Interestingly, the DMC1-mediated homologous pairing was only slightly inhibited by nucleosomes (Fig. 2b, lanes 8–11, and 2c). Taken together with the results presented in Fig. 1c–e, these results indicated that the presence of nucleosomes minimally affects the DMC1-mediated homologous pairing in both *cis* and *trans*, but substantially inhibits the RAD51-mediated homologous pairing in chromatin.

### The DMC1-ssDNA complex, but not the RAD51-ssDNA complex, disfavors nucleosome binding

We suspected that RAD51, but not DMC1, may be unable to overcome the nucleosome barrier because of its strong interaction with the nucleosome. To test this, we reconstituted nucleosomes and purified them by 6% polyacrylamide gel electrophoresis, using a Prep Cell apparatus (Bio-Rad) (Supplementary Fig. 3). The purified nucleosomes contained a 145 base-pair DNA, which was entirely wrapped around the histone octamer (without linker DNA segments).

**Figure 3:**
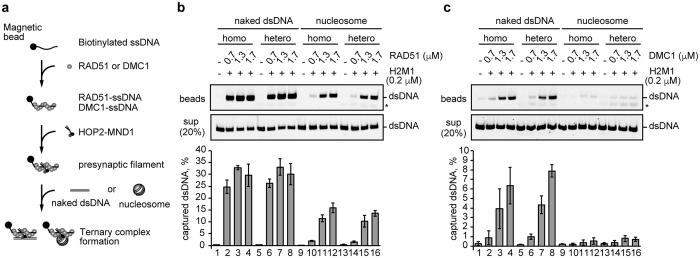
Ternary complex formation by the RAD51-ssDNA and DMC1-ssDNA complexes with a mono-nucleosome. (**a**) Scheme of the ternary complex formation assay. (**b, c**) RAD51 or DMC1 (0.7, 1.3, and 1.7 μM) was incubated with ssDNA-conjugated magnetic beads (final 5 μM in nucleotides). A heterologous poly dT 80-mer or a homologous ssDNA 80-mer was used as the ssDNA substrate. HOP2-MND1 (denoted as H2M1) was then added to the reaction mixtures. After a 5 min incubation, naked dsDNA (lanes 1–8) or mono-nucleosomes (lanes 9–16) were added to each reaction mixture. The naked and nucleosomal dsDNA concentrations were 10 μM in nucleotides. The naked or nucleosomal dsDNA captured by the RAD51-ssDNA or DMC1-ssDNA complex was treated with SDS and proteinase K, and the samples were subjected to non-denaturing polyacrylamide gel electrophoresis (top panel). The asterisk indicates poly dT 80-mer ssDNA. The naked and nucleosomal dsDNAs in the unbound fractions were also treated with SDS and proteinase K, and the samples (20%) were analyzed by non-denaturing polyacrylamide gel electrophoresis (middle panel). Bands were visualized by SYBR Gold staining. The reactions in lanes 1, 5, 9, and 13 were performed in the absence of RAD51 and DMC1. The average values of three independent experiments are shown in the bottom panel, with the SD values. Panels (**b**,**c**) represent experiments with RAD51 and DMC1, respectively.

We then performed the nucleosome binding assay with the RAD51-ssDNA and DMC1-ssDNA complexes, to evaluate their ternary complex formation activities (Fig. 3a). During the homologous recombination processes, the ternary complex containing ssDNA, dsDNA (or nucleosomal DNA), and RAD51 or DMC1 is formed in a DNA sequence-independent manner. The homologous DNA sequence between ssDNA and dsDNA (or nucleosomal dsDNA) is then aligned within the ternary complex, just before the strand invasion step. We prepared the RAD51-ssDNA and DMC1-ssDNA complexes with the ssDNA 80-mers conjugated to magnetic beads (Fig. 3a). A poly-deoxyribothymidine (dT) 80-mer and a ssDNA 80-mer containing the homologous sequence to the nucleosomal dsDNA were used as the heterologous and homologous ssDNAs, respectively. The ternary complex formation was monitored by determining the amounts of nucleosomal dsDNA captured by the RAD51-ssDNA or DMC1-ssDNA complex on the magnetic beads (Fig. 3a). The reactions were conducted in the presence of HOP2-MND1.

In this assay, the RAD51-ssDNA and DMC1-ssDNA complexes both captured the naked dsDNA in homology-independent manners, although the RAD51-ssDNA complex exhibited higher dsDNA binding activity than the DMC1-ssDNA complex (Fig. 3b,c, lanes 1–8). This suggests that, in the dsDNA binding, the turnover rate of the DMC1-ssDNA complex may be faster than that of the RAD51-ssDNA complex. The faster turnover rate may stochastically enhance the frequency of homologous pairing between ssDNA and dsDNA, as shown in Fig. 1c,d (lane 4). The RAD51-ssDNA complex also captured a substantial amount of the nucleosomes, irrespective of the homologous and heterologous ssDNAs (Fig. 3b, lanes 9–16). In contrast, we found that the nucleosome binding activity of the DMC1-ssDNA complex was quite low (Fig. 3c, lanes 9–16). These results indicated that the RAD51-ssDNA complex may be trapped by nucleosomes, but the DMC1-ssDNA complex is able to elude nucleosomes.

### The DMC1-ssDNA complex specifically targets the nucleosome-depleted dsDNA regions of the nucleosome array

We next tested whether the DMC1-ssDNA complex specifically targets the nucleosome-depleted dsDNA regions in chromatin. To do so, we prepared tri-nucleosome and di-nucleosome arrays. For the tri-nucleosome array, three purified mono-nucleosomes were ligated with 25 base-pair linker DNAs (Supplementary Fig. 4). For the di-nucleosome array, two purified nucleosomes were ligated with a dsDNA fragment, instead of the center nucleosome, resulting in a 195 base-pair nucleosome-depleted dsDNA region between the two nucleosomes (Supplementary Fig. 4). Both the length and sequence of the dsDNA were exactly the same in the tri- and di-nucleosomes. The resulting tri-nucleosomes and di-nucleosomes were further purified by native polyacrylamide gel electrophoresis with the Prep Cell apparatus (Supplementary Fig. 4).

**Figure 4:**
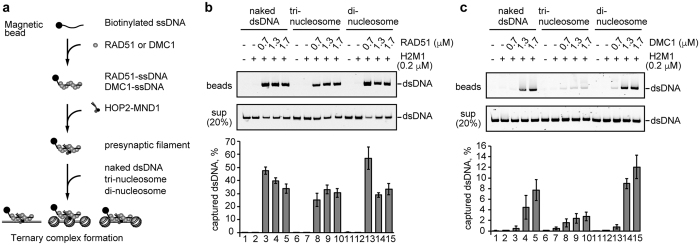
Ternary complex formation by the RAD51-ssDNA and DMC1-ssDNA complexes with nucleosome arrays. (**a**) Scheme of the ternary complex formation assay with the nucleosome arrays. (**b,c**) RAD51 or DMC1 (0.7, 1.3, and 1.7 μM) was incubated with the ssDNA (poly dT 80-mer)-conjugated magnetic beads in the presence of HOP2-MND1 (denoted as H2M1). After a 5 min incubation, naked dsDNA (lanes 1–5), tri-nucleosomes (lanes 6–10), or di-nucleosomes (lanes 11–15) were added to each reaction mixture. The naked and nucleosomal dsDNA concentrations were 10 μM in nucleotides. The naked or nucleosomal dsDNA captured by the RAD51-ssDNA or DMC1-ssDNA complex was treated with SDS and proteinase K, and the samples were subjected to non-denaturing polyacrylamide gel electrophoresis (top panel). The naked and nucleosomal dsDNAs in the unbound fractions were also treated with SDS and proteinase K, and the samples (20%) were analyzed by non-denaturing polyacrylamide gel electrophoresis (middle panel). Bands were visualized by SYBR Gold staining. The reactions in lanes 1, 6, and 11 were performed in the absence of RAD51 and DMC1. The average values of three independent experiments are shown in the bottom panel, with the SD values. Panels (**b**,**c**) represent experiments with RAD51 and DMC1, respectively.

We then performed the ternary complex formation assay with these di- and tri-nucleosome arrays (Fig. 4a). In the absence of DMC1 and RAD51, the ssDNA beads bound neither the naked dsDNA, the tri-nucleosome array, nor the di-nucleosome array (Fig. 4b,c, lanes 1, 6, and 11). HOP2-MND1 alone did not mediate the ternary complex formation with the ssDNA beads and the naked dsDNA, the tri-nucleosome array, or the di-nucleosome array (Fig. 4b,c, lanes 2, 7, and 12). In contrast, the DMC1-ssDNA and RAD51-ssDNA complexes efficiently captured the naked dsDNA in the presence of HOP2-MND1 (Fig. 4b,c, lanes 3–5). The RAD51-ssDNA complex also efficiently captured the tri-nucleosome and di-nucleosome arrays (Fig. 4b, lanes 8–10 and 13–15).

Interestingly, the DMC1-ssDNA complex exhibited quite low binding activity with the tri-nucleosome array (Fig. 4c, lanes 8–10). However, the DMC1-ssDNA complex efficiently captured the di-nucleosome array (Fig. 4c, lanes 13–15). The affinity of the DMC1-ssDNA complex was even higher for the di-nucleosome array than for the naked DNA (Fig. 4c). Therefore, these results suggested that the DMC1-ssDNA complex, but not the RAD51-ssDNA complex, possesses the ability to target the nucleosome-depleted dsDNA regions in chromatin.

### Histone tails reduce the nucleosome binding of the DMC1-ssDNA complex

**Figure 5:**
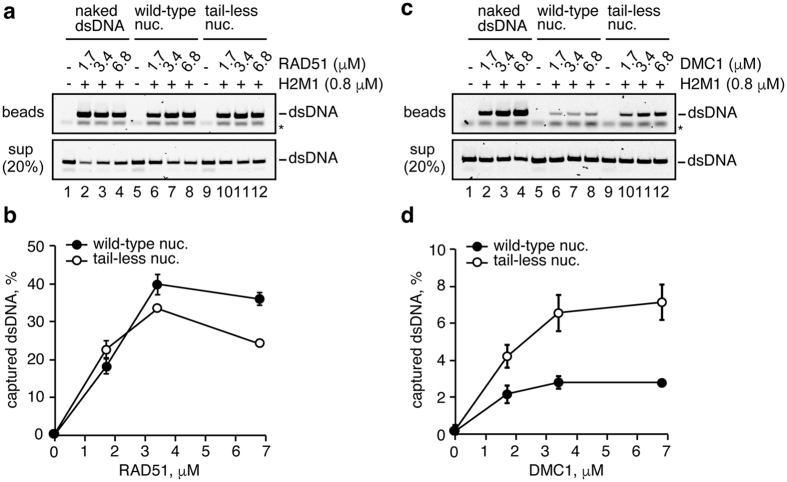
Ternary complex formation by the RAD51-ssDNA and DMC1-ssDNA complexes with the nucleosome lacking the N-terminal histone tails. (**a**) RAD51 (1.7, 3.4, and 6.8 μM) was incubated with the ssDNA-conjugated magnetic beads (final 20 μM in nucleotides). A heterologous poly dT 80-mer was used as the ssDNA substrate. HOP2-MND1 (denoted as H2M1) was then added to the reaction mixtures. After a 5 min incubation, naked dsDNA (lanes 1–4), wild-type mono-nucleosomes (lanes 5–8), or all tailless mono-nucleosomes (lanes 9–12) were added to each reaction mixture. The naked and nucleosomal dsDNA concentrations were 10 μM in nucleotides. The naked or nucleosomal dsDNA captured by the RAD51-ssDNA or DMC1-ssDNA complex was treated with SDS and proteinase K, and the samples were subjected to non-denaturing polyacrylamide gel electrophoresis (top panel). The asterisk indicates poly dT 80-mer ssDNA. The naked and nucleosomal dsDNAs in the unbound fractions were also treated with SDS and proteinase K, and the samples (20%) were analyzed by non-denaturing polyacrylamide gel electrophoresis (middle panel). Bands were visualized by SYBR Gold staining. The reactions in lanes 1, 5, and 9 were performed in the absence of RAD51 and DMC1. (**b**) Graphic representation of the experiments shown in panel (**a**). The amounts of the ternary complex formation are plotted against the RAD51 concentration. The average values of three independent experiments are shown with the SD values. (**c**) DMC1 (1.7, 3.4, and 6.8 μM) was incubated with the ssDNA-conjugated magnetic beads (final 20 μM in nucleotides). The experiments were performed by the same procedure as in panel (**a**). (**d**) Graphic representation of the experiments shown in panel (**c**). The amounts of the ternary complex formation are plotted against the DMC1 concentration. The average values of three independent experiments are shown with the SD values.

To test the contribution of the histone tails in the nucleosome binding by the RAD51-ssDNA and DMC1-ssDNA complexes, we reconstituted the mono-nucleosome with histone mutants, tlH2A, tlH2B, tlH3.1, and tlH4, which lacked the N-terminal 9, 24, 27, and 15 amino acid residues, respectively^43^ (Supplementary Fig. 5). We then performed the nucleosome binding assay with the RAD51-ssDNA and DMC1-ssDNA complexes. Since the nucleosome binding by the RAD51-ssDNA and DMC1-ssDNA complexes was not saturated under the 1.7 μM protein concentration conditions (Fig. 3), in this assay, we employed higher RAD51 and DMC1 concentrations (1.7, 3.4, and 6.8 μM). As shown in Fig. 5a,b, the removal of the histone tails did not enhance the nucleosome binding by the RAD51-ssDNA complex. However, surprisingly, the DMC1-ssDNA complex bound more efficiently to the tail-less nucleosome than the wild-type nucleosome (Fig. 5c,d). These results indicated that the histone tails contribute to the evasion of the nucleosome binding by the DMC1-ssDNA complex, but not the RAD51-ssDNA complex.

## Discussion

In meiotic cell division I, homologous recombination occurs between homologous chromosomes in the synaptonemal complex, and plays essential roles in chiasma formation. Eukaryotes have two recombinases, DMC1 and RAD51, but the functions of these two recombinases in meiotic recombination have remained enigmatic. Bugreev *et al*. made the important discovery that RAD51 is swiftly removed from the recombination site after homologous pairing, but DMC1 is resistant to dissociation from the homologous pairing products^44^. These findings suggested that, after homologous pairing, DMC1 may remain to promote chiasma formation^45^,^46^,^47^. However, the means by which DMC1 specifically promotes homologous pairing in chromatin during meiotic cell division I have remained elusive.

**Figure 6:**
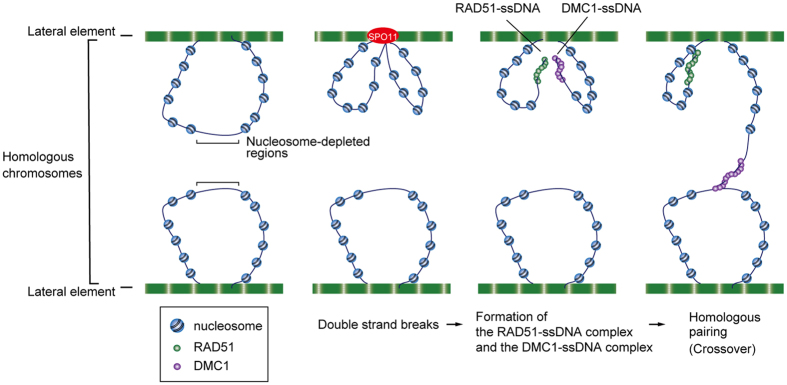
A model of DMC1-mediated initiation of meiotic homologous recombination at the defined chromosomal site. In the early leptotene stage, meiotic DSBs are induced by the SPO11 endonuclease on the chromatin loops transiently tethered to the lateral elements of the synaptonemal complex^7^,^8^,^9^,^10^. RAD51 and DMC1 are assembled on the ssDNA regions at the DSB sites. The RAD51-ssDNA complex efficiently binds to the nucleosome. Therefore, the nucleosome may impede the access of the RAD51-ssDNA complex to the homologous chromosome. In contrast, the DMC1-ssDNA complex exhibited quite low binding activity to the nucleosome, suggesting that the DMC1-ssDNA complex may access the homologous chromosome. The DMC1-ssDNA complex preferentially targets nucleosome-depleted regions, which are commonly found in the meiotic recombination hotspots.

In the present study, we found that the nucleosome binds to the RAD51-ssDNA complex, and substantially attenuates the RAD51-mediated homologous pairing in the absence of RAD54 (Figs 1, 2, 3, 4). In contrast, we found that the DMC1-ssDNA complex preferentially targets the open chromatin structure containing a nucleosome-depleted region (Figs 1, 2, 3, 4), which is commonly found in the hot spots of meiotic homologous recombination^15^,^16^,^17^,^18^. Interestingly, histone tails may function in the targeting of the nucleosome-depleted region by the DMC1-ssDNA complex (Fig. 5). These findings suggest that DMC1 may be an essential factor required for targeting the open chromatin structure, and may initiate homologous pairing at the site (Fig. 6).

Nucleosome-depleted regions are frequently found around the transcription start sites (TSSs) of active genes, and are primarily dictated by the requirements for transcriptional regulation, rather than by nucleosome remodeling factors^48^. This suggests that the TSSs with nucleosome-depleted regions may function as the target sites for the DMC1-mediated homologous pairing. Consistent with this idea, meiotic homologous recombination is initiated in intergenic regions containing TSSs in *Saccharomyces cerevisiae*^13^,^49^.

Brick *et al*.^50^ reported that, in mice, homologous recombination also occurs at loci outside TSSs. Importantly, Baker *et al*.^20^ discovered that the nucleosome-depleted regions in mouse spermatocytes are actively established by nucleosome remodeling in coordination with PRDM9 binding, which occurs concomitantly with DMC1 accumulation at the overlapping regions^50^. These *in vivo* data strongly support our conclusion that DMC1 specifically targets the nucleosome-depleted regions established at the recombination hot spots. PRDM9 trimethylates the H3K4 residue^51^, and higher H3K4me3 levels near the DSB sites have also been found in *S. cerevisiae*^52^. To establish the nucleosome-depleted regions, the nucleosome remodeling factor(s) must be recruited to the sites with the PRDM9-dependent H3K4me3. The nucleosome remodeler RAD54, which functions with RAD51, is a poor candidate because it may be inactivated during the meiotic homologous recombination process^53^,^54^ and may not target H3K4me3. The NURF complex and CHD1, which are known to target H3K4me3^55^,^56^, may function as nucleosome remodelers to establish the nucleosome-depleted regions at the homologous recombination hot spots.

In *A. thaliana* and *S. cerevisiae*, DMC1, but not RAD51, is reportedly required for the initial homologous pairing between homologous chromosomes^32^,^33^. These findings, together with our results with human proteins, consistently suggest that DMC1 may specifically function to target the programmed meiotic recombination sites, which generally contain nucleosome-depleted regions, and to promote the initial pairing between homologous chromosomes during the early stages of meiotic homologous recombination (Fig. 6).

In mouse spermatocytes, DMC1 accumulates on the recombination sites at the early stages of homologous recombination, and forms many foci on chromosomes. However, only a few crossover recombination products between homologous chromosomes were detected, suggesting that chiasma formation is controlled in a homeostatic manner^57^. One plausible explanation for the mechanism of this crossover homeostasis is that DMC1 selects the appropriate recombination sites, which are maintained as the nucleosome-depleted dsDNA regions in both the acceptor (DSB) and donor sites of homologous chromosomes. If the crossover homeostasis is regulated by the proper formation of the nucleosome-depleted region, then DMC1, but not RAD51, may play a central role for the following reasons. (i) DMC1 accumulates on many chromosomal sites, but selectively promotes homologous pairing only at the free dsDNA regions, because it may not bind to the donor dsDNA if it is wrapped in nucleosomes. (ii) RAD51 may not be available for homologous pairing in the nucleosome-depleted dsDNA regions, because the RAD51-ssDNA complex is efficiently captured by the nucleosome. These specific characteristics of DMC1 and RAD51, revealed in the present study, provide important insights to explain how meiotic homologous recombination properly occurs at the programmed sites in the synaptonemal complex, and how the number of meiotic homologous recombination sites is homeostatically maintained during homologous recombination through the chromatin structure.

## Methods

### Preparation of proteins

Human RAD51, DMC1, and the HOP2-MND1 complex were produced in *Escherichia coli* cells, and were purified by the methods described previously^37^,^58^,^59^. Human histones H2A, H2B, H3.1, H4, and those lacking the N-terminal tails were bacterially expressed, and were purified by the method described previously^43^. The histone octamer was reconstituted as described previously^60^.

### DNA

For the D-loop formation and ternary complex formation assays, high-pressure liquid chromatography-purified oligodeoxyribonucleotides (Nihon Gene Research Laboratory) were used. The ssDNA sequences used in the D-loop formation and ternary complex formation assays are listed in Supplementary Table 1. For the ternary complex formation assay with the mono-nucleosome, the 145 base-pair DNA fragment containing the Widom 601 DNA^61^ was prepared as previously described^6^. For the ternary complex formation assay with the di- and tri-nucleosome arrays, the 145 base-pair Widom 601 DNA with a 12 base-pair linker DNA at one end, and the 145 base-pair Widom 601 DNA derivative with a 13 base-pair linker DNA at both ends, were prepared by the salt-dialysis method. The DNA sequences used in the ternary complex formation assays are listed in Supplementary Table 1. All of the DNA concentrations are expressed in moles of nucleotides.

### Reconstitution of nucleosome arrays for the D-loop formation assay

Nucleosome arrays were reconstituted on the plasmid DNA with different ratios of histone octamers per 200 base pairs of donor DNA (R values = 0.4, 0.6, 0.8, 1.0), by the salt dialysis method^62^.

### Reconstitution of the di- and tri-nucleosome arrays

Three mono-nucleosomes with different sticky ends, 601a, 601b, and 601c, were reconstituted by salt dialysis, and were purified by 6% polyacrylamide gel electrophoresis, using a Prep Cell apparatus (Bio-Rad). The tri-nucleosome array was prepared by the ligation of these 601a, 601b, and 601c mono-nucleosomes. The di-nucleosome array was prepared by the ligation of the 601a mono-nucleosome, the DNA fragment containing the 601b sequence, and the 601c mono-nucleosome. The ligation reaction was conducted with T4 DNA ligase (Nippon Gene), and the resulting nucleosome arrays were further purified by 4% polyacrylamide gel electrophoresis, using a Prep Cell apparatus.

### Homologous pairing assay in chromatin

RAD51 or DMC1 (0.4 μM) was incubated with the ^32^P-labeled 5*S* 70-mer single-stranded oligonucleotide (1 μM in nucleotides) at 37 °C for 5 min, in a reaction buffer containing 24 mM HEPES (pH 7.5), 1 mM Tris-HCl, 35 mM NaCl, 45 mM KCl, 0.16 mM EDTA, 1 mM DTT, 0.4 mM 2-mercaptoethanol, 2% glycerol, 1 mM MgCl_2_, 1 mM ATP, and 100 μg/ml BSA. An aliquot (1 μl) of HOP2-MND1 (final 25 nM) was then added, and the samples were further incubated at 37 °C for 5 min. Subsequently, 1 μl of the naked dsDNA (final 30 μM in nucleotides) or nucleosomal dsDNA (final 30 μM in nucleotides) was added. The reaction mixtures were further incubated at 37 °C for 10 min, and the reactions were stopped by the addition of 2 μl of stop solution, containing SDS (0.2%) and proteinase K (1.4 mg/ml, Roche Applied Science). The resulting DNA products were analyzed by 1% agarose gel electrophoresis, in 1× TAE buffer at 4 V/cm for 2 h. The gels were dried and exposed to an imaging plate. The gel images were visualized using an FLA-7000 imaging analyzer (Fujifilm), and the band intensities were quantitated with the Multi Gauge software (Fujifilm).

### Competitive homologous pairing assay

RAD51 or DMC1 (0.4 μM) was incubated with the ^32^P-labeled 5*S* 70-mer single-stranded oligonucleotide (1 μM in nucleotides) at 37 °C for 5 min, in a reaction buffer containing 24 mM HEPES (pH 7.5), 4 mM Tris-HCl, 35 mM NaCl, 45 mM KCl, 0.46 mM EDTA, 1 mM DTT, 0.4 mM 2-mercaptoethanol, 2% glycerol, 1 mM MgCl_2_, 1 mM ATP, and 100 μg/ml BSA. An aliquot (1 μl) of HOP2-MND1 (final 25 nM) was then added, and the samples were further incubated at 37 °C for 5 min. Subsequently, a 3 μl aliquot of mono-nucleosomes (final 100, 250, 500, and 750 μM in nucleotides) was added. After a 10 min incubation at 37 °C, 1 μl of naked dsDNA (final 30 μM in nucleotides) was added. The reaction mixtures were further incubated at 37 °C for 10 min. The reactions were stopped by the addition of 2 μl of stop solution, containing SDS (0.2%) and proteinase K (1.4 mg/ml, Roche Applied Science), and the DNA products were analyzed by 1% agarose gel electrophoresis, in 1× TAE buffer at 4 V/cm for 2 h. The gels were dried and exposed to an imaging plate. The gel images were visualized using an FLA-7000 imaging analyzer (Fujifilm), and the band intensities were quantitated with the Multi Gauge software (Fujifilm).

### Analysis of nucleosome occupancy by the *Eco*RI treatment assay

Nucleosomes were reconstituted on the plasmid DNA containing ten 5*S* DNA sequences, by the salt dialysis method. The nucleosomal or naked plasmid DNA (98 ng) was digested with *Eco*RI (10 U, TOYOBO) at 37 °C for 1 h. The samples were then subjected to 6% non-denaturing polyacrylamide gel electrophoresis in 0.5× TBE buffer. After electrophoresis, the DNA bands were visualized by ethidium bromide staining, using a luminescent image analyzer (LAS-4000), and the band intensities were quantitated with the Multi Gauge software. In this analysis, the 5*S* DNA wrapped in the nucleosome migrated slowly in the gel. Therefore, the nucleosome occupancies on the 5*S* DNA sequences were estimated from the amounts of remaining nucleosome-free DNA fragments.

### MNase Assay

The purified di- and tri-nucleosome arrays (200 ng of DNA) were treated with 0, 0.05, 0.1, or 0.2 unit of MNase (Takara), in 5 μl of 22 mM Tris-HCl buffer (pH 7.5), containing 5 mM NaCl, 1 mM CaCl_2_, 1.3 mM DTT, 2.5 μg/ml BSA, and 5% glycerol. After a 3 min incubation at room temperature, the reactions were stopped by the addition of 60 μl of proteinase K solution (20 mM Tris-HCl (pH 7.5), 20 mM EDTA, 0.25% SDS, and 0.5 mg/ml proteinase K), and further incubated for 10 min. The DNA was then extracted by phenol/chloroform, and was precipitated with ethanol. The samples were fractionated by 6% PAGE in 0.2× TBE buffer.

### *Hha*I Digestion Assay

The naked dsDNA, the di-nucleosome array, and the tri-nucleosome array (200 ng of DNA) were treated with *Hha*I (10 units) at 37 °C for 1 h. The reactions were stopped by the addition of 60 μl of proteinase K solution, and further incubated at room temparature for 10 min. The DNA was purified by phenol/chloroform extraction, and was precipitated with ethanol. The DNA fragments were fractionated by 6% PAGE in 0.5× TBE buffer. The bands were visualized by ethidium bromide staining.

### Ternary complex formation assay with the mono-nucleosome

RAD51 or DMC1 (0.7, 1.3, 1.7, 3.4, and 6.8 μM) was incubated with 5′-biotinylated 601 ssDNA or 5′-biotinylated poly-dT ssDNA (80-mer, 5 μM in nucleotides) conjugated to magnetic streptavidin beads, at 37 °C for 5 min, in a reaction buffer containing 24 mM HEPES (pH 7.5), 2 mM Tris-HCl (pH 7.5), 55 mM NaCl, 25 mM KCl, 0.17 mM EDTA, 1 mM DTT, 0.4 mM 2-mercaptoethanol, 2% glycerol, 1 mM MgCl_2_, 1 mM ATP, 0.01% Nonidet P-40, and 100 μg/ml BSA. An aliquot (1 μl) of HOP2-MND1 (final 0.2 μM) was then added, and the samples were further incubated at 37 °C for 5 min. For the experiments with tail-less histones (and positive control experiments with wild-type histones), the reactions were conducted under the same conditions except for 5′-biotinylated poly-dT ssDNA (80-mer, 20 μM in nucleotides) and HOP2-MND1 (final 0.8 μM). Subsequently, 1 μl of the naked dsDNA (145 base pairs, final 10 μM in nucleotides) or the mono-nucleosome (145 base pairs, final 10 μM in nucleotides) was added to the reaction mixture. After a 10 min incubation, the beads were pelleted, and the supernatants were transferred to fresh tubes. The supernatants were deproteinized by an incubation with 1.4 mg/ml proteinase K in the presence of 0.2% SDS, at 37 °C for 15 min. The beads were washed twice with the reaction buffer, and the naked dsDNA or nucleosomal dsDNA copelleted by the ssDNA beads was recovered by a treatment with 0.2% SDS and 1.4 mg/ml proteinase K, at 37 °C for 15 min. The dsDNA samples were then fractionated by 6% polyacrylamide gel electrophoresis in 0.5× TBE buffer. For reference, 20% of the dsDNA recovered from each supernatant was fractionated by 6% polyacrylamide gel electrophoresis. The bands were visualized by SYBR Gold staining (Invitrogen) using a luminescent image analyzer (LAS-4000, Fujifilm), and the band intensities were quantitated with the Multi Gauge software.

### Ternary complex formation assay with the nucleosome arrays

RAD51 or DMC1 (0.7, 1.3, and 1.7 μM) was incubated with 5′-biotinylated poly-dT ssDNA (80-mer, 5 μM in nucleotides) conjugated to magnetic streptavidin beads, at 37 °C for 5 min, in a reaction buffer containing 24 mM HEPES (pH 7.5), 2 mM Tris-HCl (pH 7.5), 55 mM NaCl, 25 mM KCl, 0.17 mM EDTA, 1 mM DTT, 0.4 mM 2-mercaptoethanol, 2% glycerol, 1 mM MgCl_2_, 1 mM ATP, 0.01% Nonidet P-40, and 100 μg/ml BSA. An aliquot (1 μl) of HOP2-MND1 (final 0.2 μM) was then added, and the samples were further incubated at 37 °C for 5 min. Subsequently, 1 μl of the naked dsDNA (485 base pairs, final 10 μM in nucleotides), the di-nucleosome array (485 base pairs, final 10 μM in nucleotides), or the tri-nucleosome array (485 base pairs, final 10 μM in nucleotides) was added to the reaction mixture. After a 10 min incubation, the beads were pelleted, and the supernatants were transferred to fresh tubes. The supernatants were deproteinized by an incubation with 1.4 mg/ml proteinase K in the presence of 0.2% SDS, at 37 °C for 15 min. The beads were washed twice with the reaction buffer, and the naked dsDNA or nucleosomal dsDNA that copelleted with the ssDNA beads was recovered by a treatment with 0.2% SDS and 1.4 mg/ml proteinase K, at 37 °C for 15 min. The dsDNAs were then fractionated by 6% polyacrylamide gel electrophoresis in 0.5× TBE buffer. For reference, 20% of the dsDNA recovered from each supernatant was fractionated by 6% polyacrylamide gel electrophoresis. The bands were visualized by SYBR Gold staining (Invitrogen) using a luminescent image analyzer (LAS-4000, Fujifilm), and the band intensities were quantitated with the Multi Gauge software.

## Additional Information

**How to cite this article**: Kobayashi, W. *et al*. Chromatin architecture may dictate the target site for DMC1, but not for RAD51, during homologous pairing. *Sci. Rep.* **6**, 24228; doi: 10.1038/srep24228 (2016).

## Supplementary Material

Supplementary Information
