# Supplementary material for: Chromatin architecture may dictate the target site for DMC1, but not for RAD51, during homologous pairing
Source: Sci Rep. 2016 Apr 7;6:24228. doi: 10.1038/srep24228 (PMC4823753; doi:10.1038/srep24228)
Supplement: Supplementary Information [file srep24228-s1.pdf]

# Chromatin architecture may dictate the target site for DMC1, but not for RAD51, during homologous pairing

Wataru Kobayashi[1], Motoki Takaku[1, 4], Shinichi Machida[1], Hiroaki Tachiwana[1], Kazumitsu Maehara[2], Yasuyuki Ohkawa[2], and Hitoshi Kurumizaka[1, 3*]

[1] Laboratory of Structural Biology, Graduate School of Advanced Science & Engineering, Waseda University, 2-2 Wakamatsu-cho, Shinjuku-ku, Tokyo 162-8480, Japan.
[2] Division of Transcriptomics, Medical Institute of Bioregulation, Kyushu University, Fukuoka, 812-8582, Japan.
[3] Institute for Medical-oriented Structural Biology, Waseda University, 2-2 Wakamatsu-cho, Shinjuku-ku, Tokyo 162-8480, Japan.

[4] Present address: Epigenetics and Stem Cell Biology Laboratory, National Institute of Environmental Health Sciences, Research Triangle Park, North Carolina, 27709, USA.

*To whom correspondence should be addressed. E-mail: kurumizaka@waseda.jp (H.K.)

Supplementary Figure 1-5
Supplementary Table 1

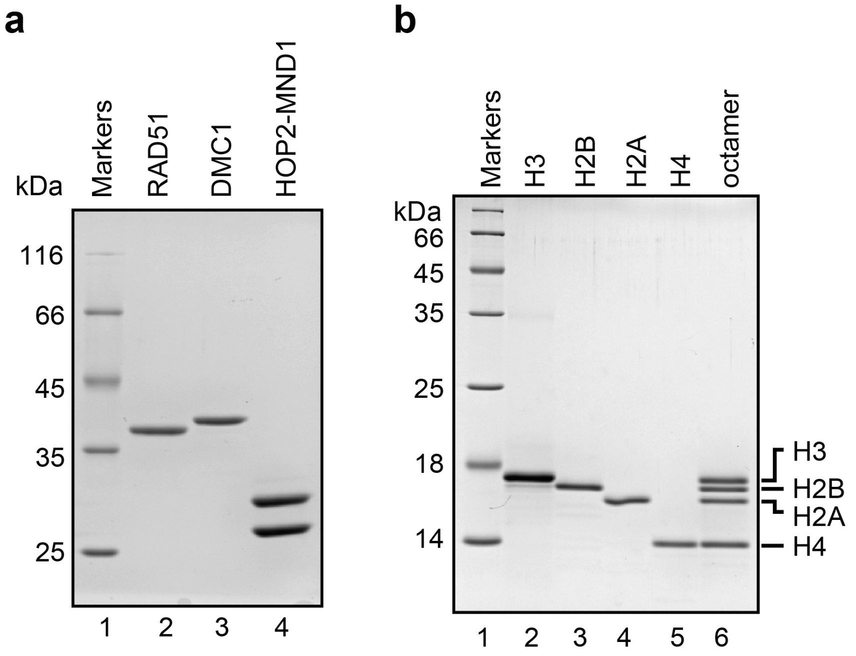


**Supplementary Figure 1** Preparation of proteins. (**a**) Human RAD51, DMC1, and the HOP2-MND1 complex. Purified recombinant RAD51 (0.75 μg), DMC1 (0.75 μg), and the HOP2-MND1 complex (1.5 μg) were analyzed by 12% SDS-PAGE with Coomassie Brilliant Blue staining. (**b**) Human histones and histone octamers. Purified histones H2A, H2B, H3.1, H4, and reconstituted histone octamers were analyzed by 18% SDS-PAGE with Coomassie Brilliant Blue staining.

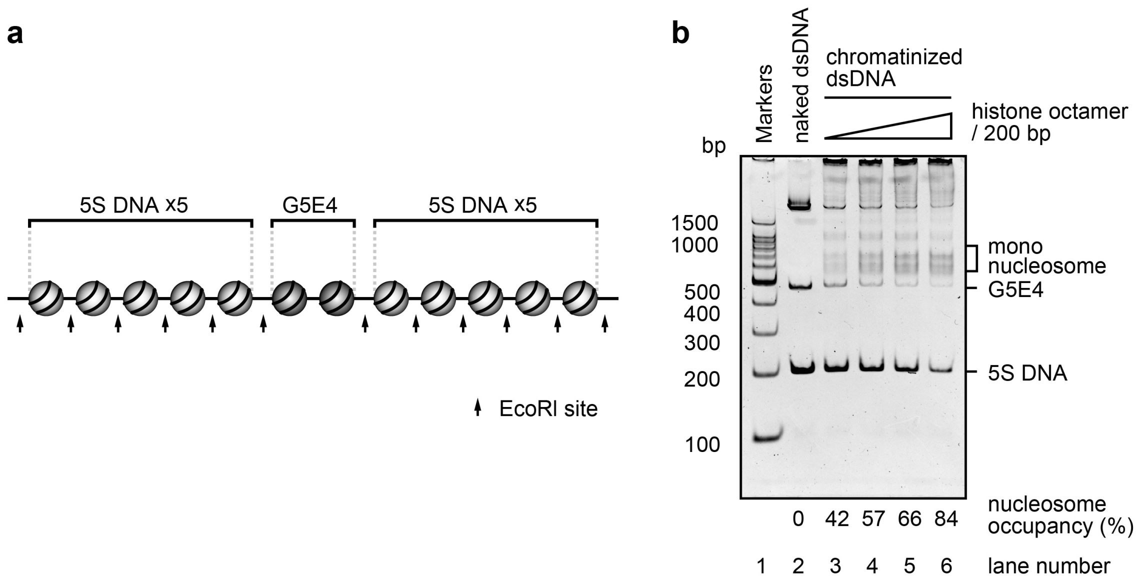


**Supplementary Figure 2** Preparation of nucleosome arrays for the D-loop formation assay. (**a**) Schematic representation of the nucleosome array. The two nucleosomes on the G5E4 sequence are located between five 5*S* DNA nucleosome arrays. The arrows indicate the *Eco*RI site between the tandem 5*S* DNAs. (**b**) The *Eco*RI digestion analysis. The reconstituted nucleosome arrays were treated with *Eco*RI, which digests the linker DNA regions between the 5*S* DNA nucleosomes. After the *Eco*RI treatment, the resulting 5*S* DNA nucleosomes were separated from the nucleosome-free 5*S* DNA fragments on a non-denaturing polyacrylamide gel, and the nucleosome occupancies on the 5*S* sequences were estimated by comparisons of the amounts of the nucleosome-free 5*S* DNA fragments with the amounts of DNA fragments from the *Eco*RI-treated naked dsDNA.

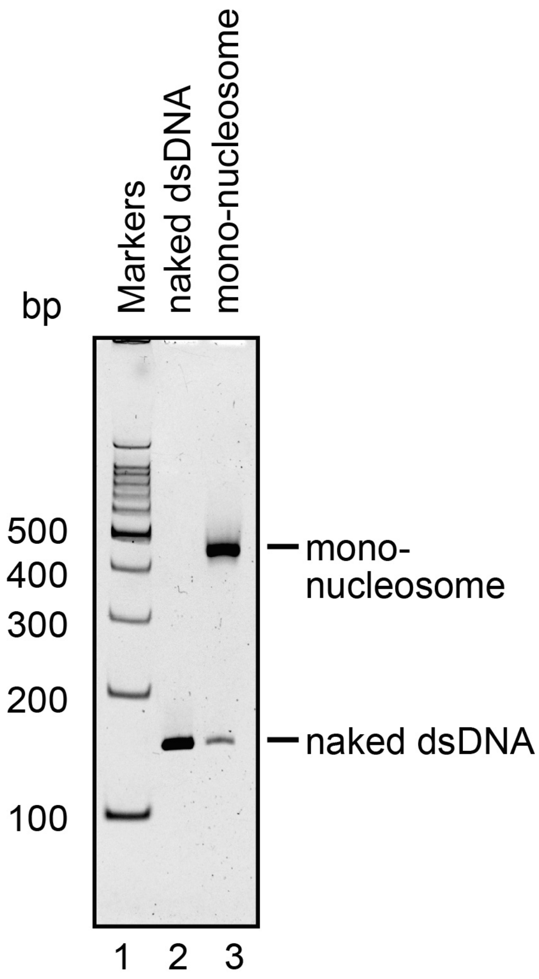


**Supplementary Figure 3** Preparation of the mono-nucleosome. The nucleosome was reconstituted with the 145 base-pair Widom 601 sequence and the histone octamer, by the salt-dialysis method. The reconstituted nucleosome was further purified by 6% polyacrylamide gel electrophoresis, using a Prep Cell apparatus. The purified nucleosome was analyzed by 6% PAGE with EtBr staining. Lane 1 indicates DNA markers. Lanes 2 and 3 represent naked dsDNA and the mono-nucleosome, respectively.

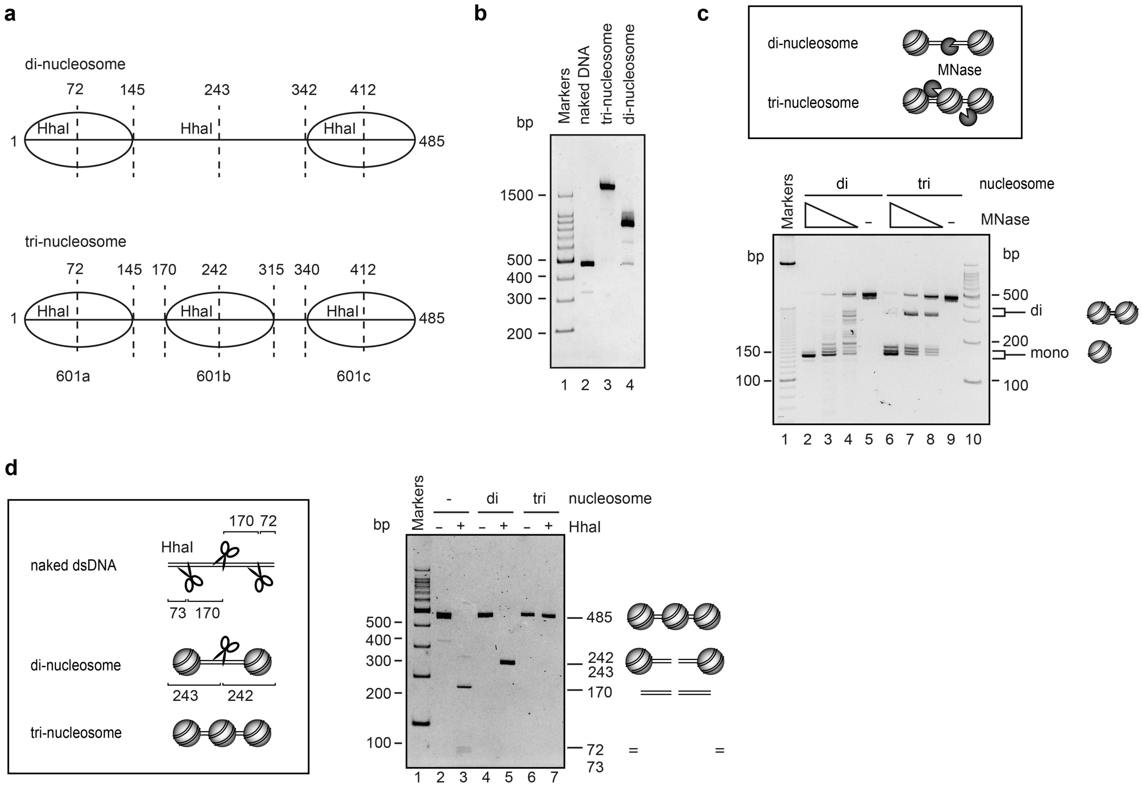


**Supplementary Figure 4** Preparation of the di- and tri-nucleosome arrays. (**a**) Schematic representations of the di- and tri-nucleosomes. Ellipses represent the positions of the nucleosomes. The *Hha*I sites are indicated. (**b**) Purified nucleosome arrays. Di- and tri-nucleosomes were purified by non-denaturing polyacrylamide gel electrophoresis. The naked dsDNA (485 base pairs) and the purified di- and tri-nucleosomes were analyzed by 4% non-denaturing polyacrylamide gel electrophoresis. Bands were visualized by EtBr staining. (**c**) MNase analysis of the di- and tri-nucleosome arrays. The di- and tri-nucleosome arrays were treated with 0 U (lanes 5 and 9), 0.05 U (lanes 4 and 8), 0.1 U (lanes 3 and 7), and 0.2 U (lanes 2 and 6) of MNase, and the DNAs extracted from the nucleosomes were analyzed by non-denaturing polyacrylamide gel electrophoresis. Bands were visualized by EtBr staining. Lanes 1 and 10 indicate DNA markers. (**d**) *Hha*I digestion analysis of nucleosome arrays. The naked dsDNA, the di-nucleosome array, and the tri-nucleosome array were incubated with (lanes 3, 5, and 7) or without (lanes 2, 4, and 6) *Hha*I, and the resulting DNA fragments were fractionated by non-denaturing polyacrylamide gel electrophoresis. Bands were visualized by EtBr staining. Lane 1 indicates DNA markers.

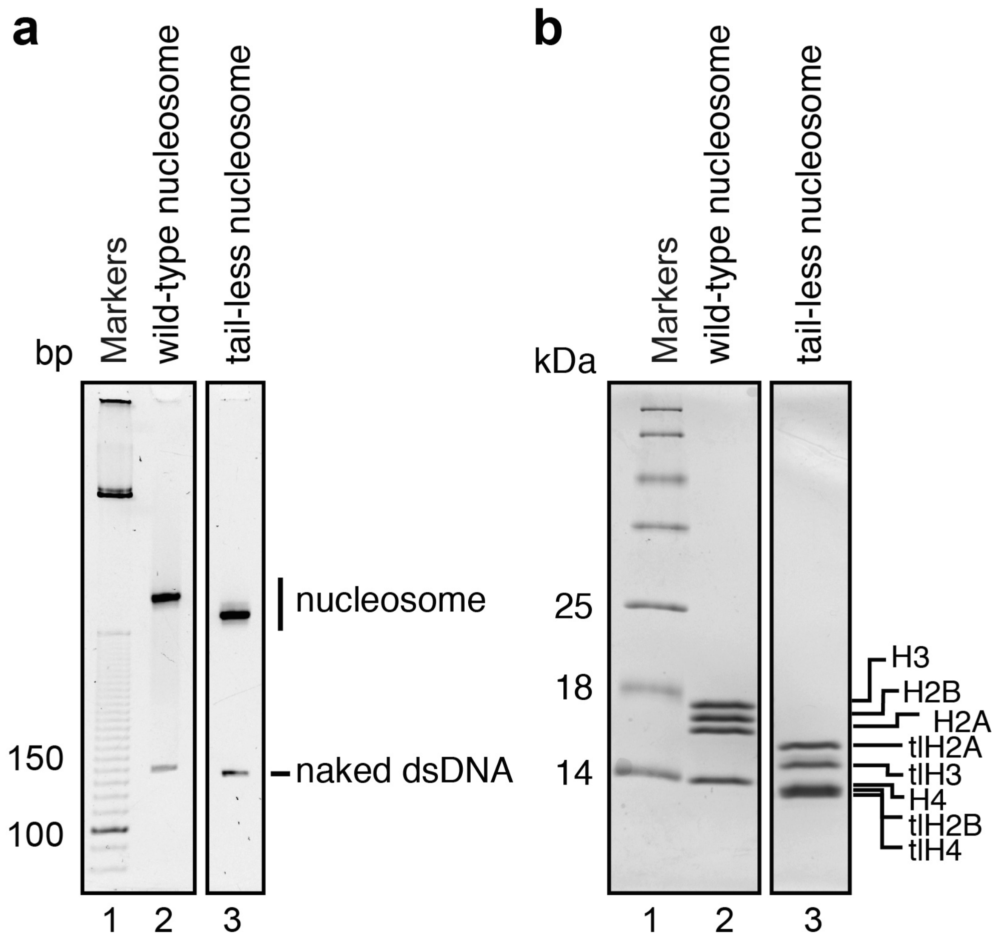


**Supplementary Figure 5** Preparation of wild-type and tail-less nucleosomes. (**a**) The reconstituted nucleosomes were purified by 6% polyacrylamide gel electrophoresis, using a Prep Cell apparatus. The purified nucleosomes were analyzed by 6% PAGE with EtBr staining. Lane 1 indicates DNA markers. Lanes 2 and 3 represent wild-type nucleosome and tail-less nucleosome, respectively. (**b**) Histone compositions of the nucleosomes were analyzed by 20% SDS-PAGE with Coomassie Brilliant Blue staining.

**Supplemental Table 1. Sequences of the DNA substrates**

| | | |
|---|---|---|
| 1 | 5'biotinylated 80 mer poly-dT ssDNA | TTTTT TTTTT TTTTT TTTTT TTTTT TTTTT TTTTT TTTTT TTTTT TTTTT TTTTT TTTTT TTTTT TTTTT TTTTT TTTTT |
| 2 | 5'biotinylated 80 mer homologous ssDNA | TCGTA GACAG CTCTA GCACC GCTTA AACGC ACGTA CGCGC TGTCC CCCGC GTTTT AACCG CCAAG GGGAT TACTC CCTAG |
| 3 | 5S 70 mer ssDNA | CCGGT ATATT CAGCA TGGTA TGGTC GTAGG CTCTT GCTTG ATGAA AGTTA AGCTA TTTAA AGGGT CAGGG |
| 4 | 601a dsDNA | ATCAG AATCC CGGTG CCGAG GCCGC TCAAT TGGTC GTAGA CAGCT CTAGC ACCGC TTAAA CGCAC GTACG CGCTG TCCCC CGCGT TTTAA CCGCC AAGGG GATTA CTCCC TAGTC TCCAG GCACG TGTCA GATAT ATACA TCGAT TGGAT AGGCC C |
| 5 | 601b dsDNA | GGACG GCCTG GATAA TCAGA ATCCC GGTGC CGAGG CCGCT CAATT GGTCG TAGAC AGCTC TAGCA CCGCT TAAAC GCACG TACGC GCTGT CCCCC GCGTT TTAAC CGCCA AGGGG ATTAC TCCCT AGTCT CCAGG CACGT GTCAG ATATA TACAT CGATT GGATA GGCCC CAA |
| 6 | 601c dsDNA | CGGCC TGGAT AATCA GAATC CCGGT GCCGA GGCCG CTCAA TTGGT CGTAG ACAGC TCTAG CACCG CTTAA ACGCA CGTAC GCGCT GTCCC CCGCG TTTTA ACCGC CAAGG GGATT ACTCC CTAGT CTCCA GGCAC GTGTC AGATA TATAC ATCGA T |
